# Loss of Epithelial Homeostasis Driven by TMBIM1 Depletion via E-Cadherin Junction Disassembly

**DOI:** 10.3390/ijms27021090

**Published:** 2026-01-22

**Authors:** Zhenning Sun, Lei Zhang, Junxia Qi, Min Jiang, Shan Jiang, Zining Zhu, Yanxuan Ling, Xiaobin Wang, Juxue Li

**Affiliations:** 1 School of Biological Science and Medical Engineering, Southeast University, Nanjing 210096, China; 2 Jiangsu Provincial Key Laboratory of Molecular Targets and Intervention of Metabolic Disease, Nanjing Medical University, Nanjing 211166, China; 3 Laboratory Animal Centre, Southeast University, Nanjing 210096, China; 4 School of Medicine, Southeast University, Nanjing 210009, China

**Keywords:** TMBIM1, COAD, cell adhesion, E-cadherin, MSI

## Abstract

Mounting evidence from large-scale association studies has identified transmembrane BAX inhibitor motif-containing 1 (*TMBIM1*) as a promising candidate gene in colorectal cancer (CRC) pathogenesis. Our clinical analysis confirmed this association, demonstrating significantly reduced TMBIM1 expression in human colon cancer tissues. To elucidate its functional role, we employed complementary experimental approaches across different cellular contexts. In normal colonic epithelial cells (NCM460), TMBIM1 deficiency triggered distinct morphological changes and suppressed cellular growth. Conversely, in malignant HCT-116 cells, TMBIM1 knockdown paradoxically enhanced proliferation and other pro-tumorigenic characteristics, suggesting context-dependent functions. Transcriptomic profiling via RNA-seq revealed that TMBIM1 suppression enhances cell viability, and the specific mutational background of HCT-116 cells appears to exploit the consequent loss of E-cadherin to further drive progression. Mechanistic investigations further identified E-cadherin (*CDH1*) as a key downstream effector, showing significant down-regulation following TMBIM1 knockdown. We therefore define a context-dependent tumor-suppressive mechanism for TMBIM1, wherein its loss in MSI-H cells promotes tumorigenesis via E-cadherin suppression and the consequent loss of epithelial integrity.

## 1. Introduction

Colon adenocarcinoma (COAD) represents a major global health burden, with high incidence and mortality rates worldwide. It ranks as the third most commonly diagnosed malignancy after lung and breast cancer and is the second leading cause of cancer-related deaths following lung cancer [1]. The pathogenesis of COAD involves a complex interplay of genetic alterations [2], environmental factors [3,4], dietary habits [5,6], and socioeconomic influences [7,8], which collectively contribute to colonic epithelial transformation, chronic inflammation, and ultimately carcinogenesis. Among genetic factors, loss-of-function mutations and dysregulation of specific genes have been increasingly implicated in colon cancer development. Genomic studies have linked the transmembrane BAX inhibitor motif-containing 1 gene (*TMBIM1)* to colorectal cancer susceptibility. For instance, it was identified as a risk variant in patients with serrated polyposis syndrome (SPS) [9], and a meta-analysis of multiple genome-wide association studies (GWASs) further established TMBIM1 as a susceptibility locus for COAD in Chinese populations [10]. However, the mechanistic basis of their contributions to COAD pathogenesis remains incompletely understood.

*TMBIM1* (transmembrane BAX inhibitor motif-containing 1), initially identified as *RECS1* (responsive to centrifugal force and shear stress 1), is a shear stress-responsive gene [11]. It belongs to the TMBIM family, whose members (TMBIM1–6) are known for their anti-apoptotic functions [12,13,14,15]. Specifically, TMBIM1 has been shown to inhibit Fas-mediated apoptosis [16,17]. However, a recent report suggests a context-dependent pro-apoptotic role for TMBIM1, demonstrating that it acts as a pH-regulated calcium channel that promotes lysosomal membrane permeabilization, facilitates BCL2-Associated X Protein (BAX) translocation to lysosomes, and induces apoptosis in response to cytotoxic stimuli [12,18]. Beyond its role in apoptosis, TMBIM1 has been implicated in various metabolic processes. It modulates vascular remodeling in aging mice, protects against non-alcoholic liver disease by promoting lysosomal degradation, and ameliorates obesity-associated pathologies such as cardiomyopathy through attenuation of oxidative stress and inflammation [11,19,20]. Interestingly, TMBIM1 acts as an inhibitor of adipogenesis, and its loss exacerbates adipocyte hyperplasia while paradoxically improving metabolic disease in obese models [19,21]. These pleiotropic functions strongly suggest that TMBIM1 may play a unique and context-dependent role in colon carcinogenesis, yet its specific mechanistic contributions to colorectal cancer progression remain poorly understood.

To address this gap, we investigated the expression profile of TMBIM1 in clinical COAD samples compared with adjacent non-tumor tissues. We further employed knockdown and over-expression strategies in colon cancer cell lines to assess the functional impact of TMBIM1 on malignant phenotypes. RNA sequencing analysis following TMBIM1 modulation was performed to identify downstream transcriptional targets. Among these, E-cadherin, a well-established marker in COAD, was significantly down-regulated upon TMBIM1 knockdown. Collectively, our integrated analyses suggest that in MSI colon cancer cells, TMBIM1 modulates malignant phenotypes, in part through regulating E-cadherin expression and thereby influencing epithelial adhesion.

## 2. Results

### 2.1. TMBIM1 Is Highly Expressed in Normal Colonic Epithelium and Down-Regulated in Colorectal Cancer

**Figure 1:**
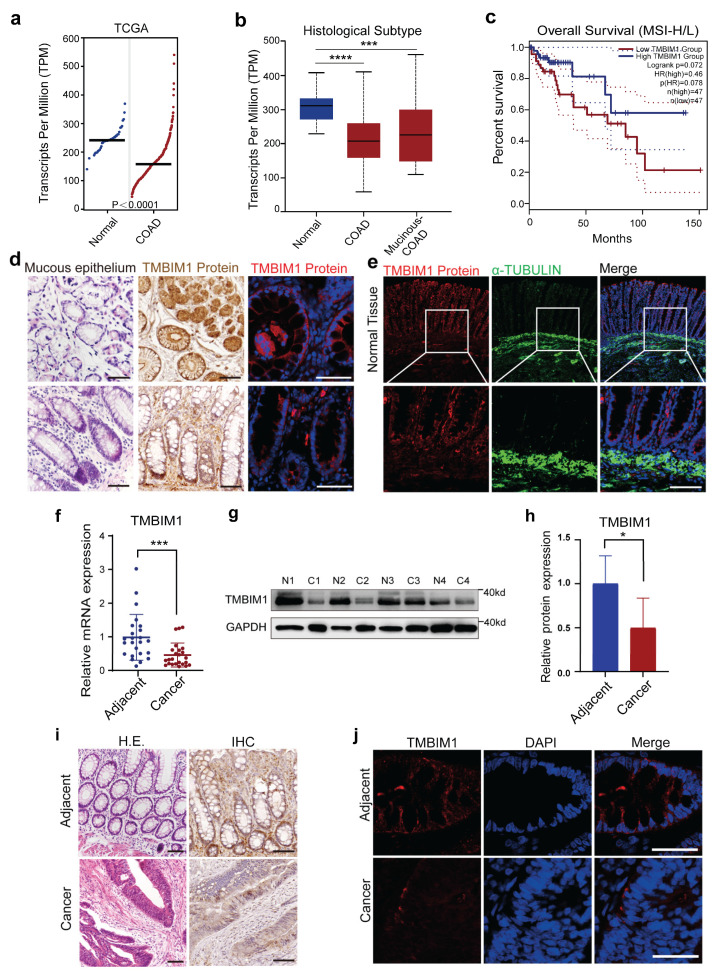
Expression of TMBIM1 in colon cancer and normal tissues. (**a**) Analysis of *TMBIM1* expression in healthy individuals and COAD patients based on TCGA data (healthy individuals, n = 41; COAD, n = 275; *p* < 0.0001). (**b**) *TMBIM1* expression across normal tissues, COAD, and mucinous COAD subtypes (n = 41, 243, 37). Normal vs. COAD, *p* < 0.001; Normal vs. mucinous COAD, *p* < 0.001; COAD vs. mucinous COAD, not significant. (**c**) Survival analysis stratified by TMBIM1 expression in MSI-H/L colon cancer patients. n = 47 per group, log-rank *p* = 0.072. Data normalized to *ACTB* and analyzed via GEPIA2, based on data from TCGA. Dotted lines depict the 95% confidence intervals of the survival curves. (**d**) Immunohistochemical and immunofluorescence staining of TMBIM1 in transverse and longitudinal sections of normal colon epithelium, scale bar = 50 µm. (**e**) Immunofluorescence analysis detected strong TMBIM1 signal primarily in the colonic mucosa, with specific enrichment in the crypt structures, scale bar = 50 µm. (**f**) Quantitative PCR comparison of *TMBIM1* expression in human colon cancer tissues and matched adjacent normal tissues (n = 24; *** *p* < 0.001). (**g**,**h**) Western blot analysis and quantification of TMBIM1 protein levels in patient-matched cancer (C) and normal adjacent (N) tissues (n = 8; * *p* < 0.05). (**i**) Immunohistochemical staining of TMBIM1 in colon cancer and adjacent normal tissues, scale bar = 50 µm. (**j**) Immunofluorescent staining showing TMBIM1 expression in colon cancer and adjacent normal tissues, scale bar = 50 µm. Data represent means ± SD. Statistically significant differences were determined using One-way ANOVA (**c**) or paired *t*-test (**f**,**h**). * *p* < 0.05; *** *p* < 0.001; **** *p* < 0.0001.

Our analysis reveals that TMBIM1 down-regulation is associated with worse prognosis specifically in the microsatellite instability-high/low (MSI-H/L) subtype of colorectal cancer. The initial analysis of The Cancer Genome Atlas (TCGA) database revealed that TMBIM1 expression is significantly lower in COAD and READ tissues than in normal samples (Figure 1a and Figure S1a). This differential expression pattern remained consistent across both COAD and READ histological subtypes (Figure 1b and Figure S1b). Survival analysis indicated that the prognostic impact of TMBIM1 expression depends on microsatellite instability (MSI) status. Specifically, high TMBIM1 expression did not confer a significant overall survival benefit in COAD patients of the MSS subtype (Figure S1c) or in the general READ patient cohort (Figure S1d). In contrast, it was notably associated with better short-term survival (within 50 months) specifically in the MSI-H/L subgroup of COAD patients (Figure 1c).

To confirm TMBIM1 expression in COAD, we performed immunohistochemical staining, quantitative PCR (qPCR) amplification, and Western blot (WB) protein analysis. In normal human colon tissues, we found that TMBIM1 was highly expressed in the colonic epithelium, particularly at the base of the intestinal crypts (Figure 1d,e), which harbor the stem and progenitor cells considered the cell-of-origin for many colorectal adenocarcinomas. Both qPCR and WB analyses revealed significantly lower TMBIM1 levels in colon adenocarcinoma tumor tissues (Figure 1f–h), consistent with the TCGA data. Immunohistochemical analysis further confirmed this down-regulation, with well-structured normal glands exhibiting strong TMBIM1 staining (Figure 1i), contrasting sharply with its marked reduction in architecturally disrupted tumor regions (Figure 1j). The consistent downregulation of TMBIM1 across multiple datasets and methods strongly associates it with the colon adenocarcinoma state.

### 2.2. TMBIM1 Depletion Impairs Normal Cellular Morphology in NCM460 Cells

**Figure 2:**
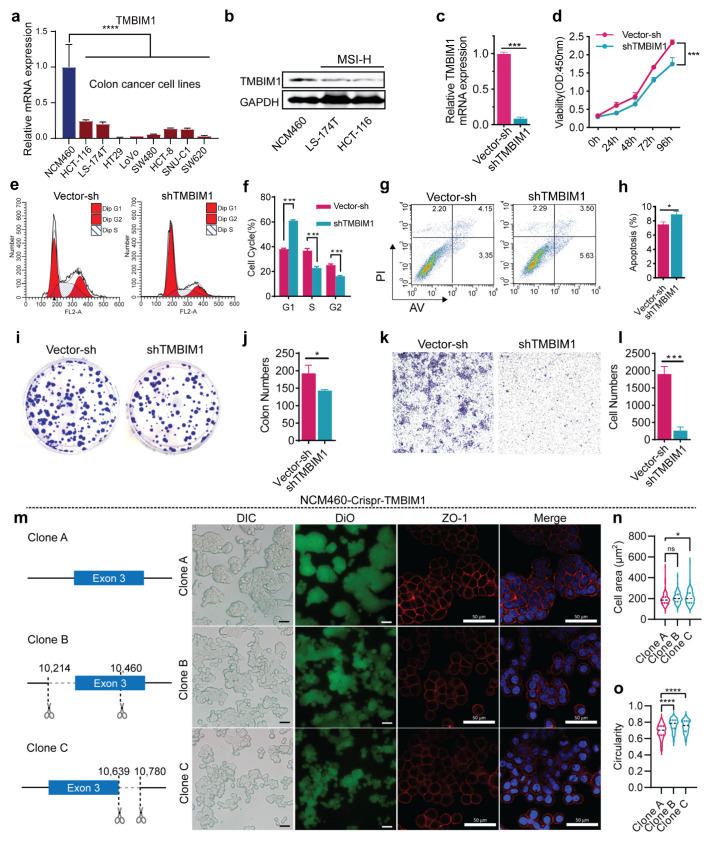
TMBIM1 knockdown in NCM460 cells inhibits cell growth and alters cell morphology. (**a**) Relative mRNA expression of *TMBIM1* in colon epithelial cells (NCM460) and colon cancer cell lines. (**b**) Schematic representation of the lentivirus-mediated TMBIM1 knockdown strategy in NCM460 cells. (**c**) Relative mRNA expression of *TMBIM1* in *TMBIM1*-knockdown NCM460 cells (shTMBIM1) and control cells (Vector-sh). (**d**) The viability of shTMBIM1 and Vector-sh cells cultured for 24, 48, 72, 96 h. (**e**,**f**) Represent results of shTMBIM1 and Vector-sh groups’ cell cycle, and statistics. n = 3. (**g**,**h**) Represent results of shTMBIM1 and Vector-sh groups’ apoptosis tests, and statistic of shTMBIM1 and Vector-sh groups. n = 3. (**i**,**j**) Represent results of shTMBIM1 and Vector-sh groups’ clone formation assays and quantitative analysis of colonies in Vector-sh and shTMBIM1 groups. (**k**,**l**) Represent results of shTMBIM1 and Vector-sh groups’ transwell migration assay, and statistic results of shTMBIM1 and Vector-sh groups. (**m**) Differential interference contrast (DIC), DiO and ZO-1 immunofluorescence staining in Clone A, B, and C cells; scale bar = 50 μm. (**n**) Violin plot showing cell area quantification of Clone A, B, and C cells, analyzed using ImageJ (v1.53c). (**o**) Violin plot showing cell circularity quantification of Clone A (n = 177 cells), Clone B (n = 120 cells), and Clone C (n = 122 cells) from three independent cultures. Data represent means ± SD. Statistical significance was determined by two-way ANOVA with Dunnett’s multiple comparisons test (**d**), one-way ANOVA with Dunnett’s multiple comparisons test (**n**,**o**), or two-tailed Student’s *t*-test (rest), ns, not significant. * *p* < 0.05; *** *p* < 0.001; **** *p* < 0.0001.

To further confirm the findings from clinical COAD samples, we examined TMBIM1 expression at both mRNA levels across colon cancer cell lines such as HCT-116, LS-174T, HT29, LoVo, SW480, HCT-8, SNU-C1, SW620 and the normal colon epithelial cell line NCM460. Strikingly, comparative analysis revealed at least a threefold higher *TMBIM1* expression in NCM460 cells versus cancer lines (Figure 2a), mirroring the clinical tissue pattern. Accordingly, TMBIM1 protein expression was substantially lower in MSI-subtype cell lines (LS-174T, HCT-116) than in NCM460 cells (Figure 2b).

This conserved down-regulation in malignant contexts prompted us to investigate TMBIM1’s functional role in carcinogenesis. We selected the NCM460 cell line as our model system due to its high endogenous TMBIM1 expression. To explore the potential role of TMBIM1 in early carcinogenesis, we first generated stable *TMBIM1*-knockdown NCM460 cells (NCM460-shTMBIM1 cells) to mimic early carcinogenic conditions (Figure 2c). The shTMBIM1 cells displayed markedly growth inhibition, as indicated by reduced cell viability (Figure 2d). Subsequent analysis of the cell cycle revealed an increase in the G1 phase and a decrease in the S and G2 phases post *TMBIM1* knockdown, indicative of cell cycle arrest at the G1/S phase checkpoint (Figure 2e,f). Additionally, flow cytometry showed increased apoptosis in NCM460-shTMBIM1 (Figure 2g,h). Colony formation assays demonstrated a decreased ability of NCM460-shTMBIM1cells to form colonies. (Figure 2i,j). Evaluation of migration ability via transwell assay revealed a significant reduction in migration upon *TMBIM1* knockdown (Figure 2k,l). These findings strongly suggest that TMBIM1 plays a role in maintaining normal metabolic and functional activities of epithelial cells.

Crystal violet staining revealed notable morphological alterations and weakened intercellular connections in NCM460-shTMBIM1 compared to controls (Figure S2a). To verify the morphologic changes induced by TMBIM1 knockdown, we utilized Crispr-Cas9 to knock out *TMBIM1* in NCM460 cells: Crispr-TMBIM1-A (normal NCM460), Crispr-TMBIM1-B (heterozygous knockout), and Crispr-TMBIM1-C (homozygote knockout) (Figure S2b–d). Immunofluorescence staining of Zonula Occludens-1 (ZO-1) showed that the characteristic continuous linear distribution of ZO-1 at cell–cell junctions was disrupted in Crispr-TMBIM1-B and -C cells compared to controls, displaying a more punctate and irregular pattern (Figure 2m). Quantitative analysis of cell area and circularity confirmed that TMBIM1 deficiency caused NCM460 cells to adopt a rounder morphology (Figure 2n,o).

Overall, it was observed that NCM460 cells exhibit high level of TMBIM1 expression, and its deficiency alters multiple cellular phenotypes including proliferation, cell cycle progression, apoptosis resistance, colony formation, migration, and morphological integrity in vitro.

### 2.3. TMBIM1 Knockdown Promotes Colon Cancer Cell Growth

**Figure 3:**
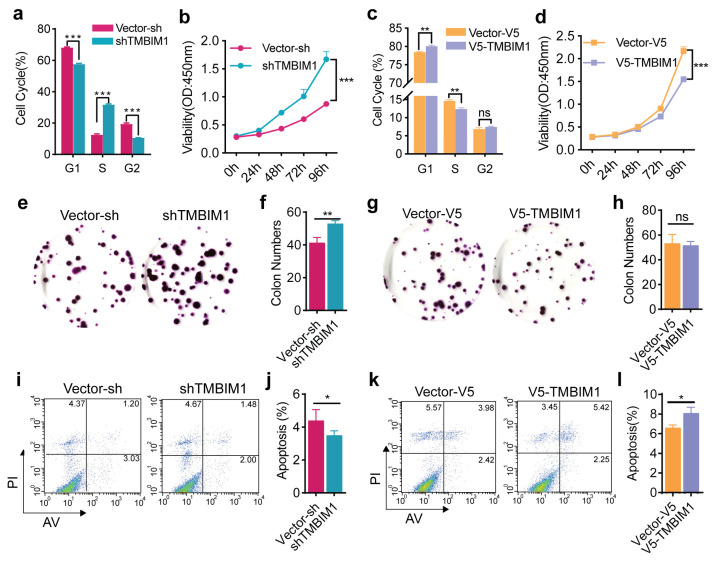
Effects of TMBIM1 on proliferation, apoptosis, and cell cycle of HCT-116 cells. (**a**) Cell cycle distribution in shTMBIM1 cells measured by flow cytometry. (**b**) CCK-8 proliferation assay of shTMBIM1 cells (n = 3 per group, measured over 0–96 h). (**c**) Cell cycle distribution in V5-TMBIM1 cells. (**d**) CCK-8 proliferation assay of V5-TMBIM1 cells (n = 3 per group, measured over 0–96 h). (**e**,**f**) Colony formation assay and quantification in shTMBIM1 cells (n = 3). (**g**,**h**) Colony formation assay and quantification in V5-TMBIM1 cells (n = 6 per group). (**i**,**j**) Apoptosis analysis by flow cytometry in shTMBIM1 cells (n = 3 per group). (**k**,**l**) Apoptosis analysis by flow cytometry in V5-TMBIM1 cells (n = 3 per group). Data represent means ± SD. Statistically significant differences were determined using two-way ANOVA with Bonferroni’s multiple comparisons test (**b**,**d**) or two-tailed student’s test (rest). ns, not significant; * *p* < 0.05; ** *p* < 0.01; *** *p* < 0.001.

Observation of the association between high TMBIM1 expression and favorable prognosis in the COAD MSI-H/L subgroup prompted the hypothesis that TMBIM1 may play an important role in the MSI subtype. To investigate TMBIM1’s role in the pathogenesis of MSI-characterized colon cancer, we genetically manipulated its expression in the COAD cell line HCT-116 through both knockdown (HCT-116-shTMBIM1) and overexpression (HCT-116-V5-TMBIM1) approaches, systematically evaluating effects on proliferation, apoptosis, and cell cycle regulation. Western blot and qPCR analyses confirmed efficient genetic perturbation, with HCT-116-shTMBIM1 achieving > 80% knockdown and HCT-116-V5-TMBIM1 showing 13-fold overexpression relative to controls (Figure S3a–d). Additionally, immunofluorescent staining of TMBIM1 protein in stable cell lines revealed that TMBIM1 is predominantly located in the cytoplasm, despite reports suggesting its secretory function (Figure S3e,f). Functional analyses demonstrated that TMBIM1 knockdown promoted oncogenic phenotypes, including elevated S-phase fraction (Figure 3a), enhanced proliferation (Figure 3b), increased colony formation (Figure 3e–f), and reduced apoptosis (Figure 3i,j), while conversely, TMBIM1 over-expression exerted tumor-suppressive effects through G1 phase prolongation (Figure 3c), growth inhibition (Figure 3d,g,h), and apoptosis induction (Figure 3k,l). To substantiate these findings in another MSI background, we examined the tumor-suppressive effect of TMBIM1 overexpression in the LS-174T cell line (another MSI colon cancer cell line). Consistently, TMBIM1 overexpression significantly inhibited oncogenic phenotypes, as evidenced by reduced colony formation capacity and an increased apoptosis rate in LS-174T-V5-TMBIM1 cells (Figure S4a–d). Together, our findings support the conclusion that TMBIM1 functions as a tumor suppressor in MSI colon cancer models by inhibiting proliferation and promoting apoptosis, highlighting its potential role in this specific cancer subtype.

### 2.4. TMBIM1 Suppresses Colon Tumor Growth In Vivo

**Figure 4:**
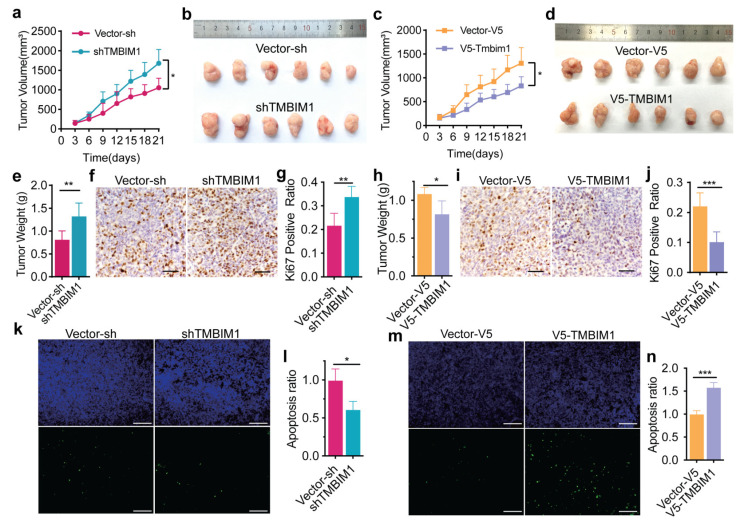
TMBIM1 impressed tumor growth of HCT-116 in vivo. (**a**) Tumor volume measurements in nude mice injected with shTMBIM1 or control cells (n = 6, * *p* < 0.05). (**b**) Representative images of excised tumors from shTMBIM1 and control groups. (**c**) Quantitative analysis of tumor weight in shTMBIM1 and control groups (n = 6, * *p* < 0.05). (**d**) Representative images of excised tumors from V5-TMBIM1 and control groups. (**e**) Tumor weight analysis of shTMBIM1 and control groups (n = 6, ** *p* < 0.01). (**f**,**g**) Ki67 immunohistochemical staining and quantification of proliferating cells in shTMBIM1 and control tumors (n = 4, ** *p* < 0.001). (**h**) Tumor weight analysis of V5-TMBIM1 and control groups (n = 6, * *p* < 0.05). (**i**,**j**) Ki67 immunohistochemical staining and quantification of proliferating cells in V5-TMBIM1 and control tumors (n = 4, *** *p* < 0.001). (**k**,**l**) TUNEL assay and quantitative analysis of apoptotic cells in shTMBIM1 and control tumors (n = 3, * *p* < 0.05), scale bar = 200 µm. (**m**,**n**) TUNEL assay and quantification of apoptosis in V5-TMBIM1 and control tumors (n = 3, *** *p* < 0.001), scale bar = 200 µm. Data represent means ± SD. Statistically significant differences were determined using two-way ANOVA with Bonferroni’s multiple comparisons test (**a**,**c**) or two-tailed student’s test (rest). * *p* < 0.05; ** *p* < 0.01; *** *p* < 0.001.

Our investigation of TMBIM1’s role in COAD progression employed a xenograft model to evaluate its tumor-modulating effects in vivo. Using stable HCT-116 cell lines with either TMBIM1 knockdown (HCT-116-shTMBIM1 cells) or overexpression (HCT-116-V5-TMBIM1 cells), we observed striking phenotypic differences in tumor growth over 14 days. Tumors derived from HCT-116-V5-TMBIM1 cells showed significant reductions in both volume and weight compared to controls (Figure 4a–d), while HCT-116-shTMBIM1 tumors displayed accelerated growth with increased mass (Figure 4e,h). Complementary immunohistochemical analyses revealed mechanistic insights: Ki67 staining demonstrated enhanced proliferation in shTMBIM1 tumors versus suppressed proliferation in HCT-116-V5-TMBIM1 tumors (Figure 4f,g,i,j), while reciprocal apoptosis patterns diminished cell death in HCT-116-shTMBIM1 tumors (Figure 4k,l) versus increased apoptosis in HCT-116-V5-TMBIM1 tumors (Figure 4m,n). These consistent in vivo findings establish that modulating TMBIM1 expression can control tumor growth in an HCT-116 xenograft model. The observed effects are associated with changes in proliferation and apoptosis. This work provides a foundation for future studies to explore the role of TMBIM1 in specific molecular subtypes of colon cancer.

### 2.5. TMBIM1 Knockdown Inhibits Cell Adhesion in HCT-116 Cells

**Figure 5:**
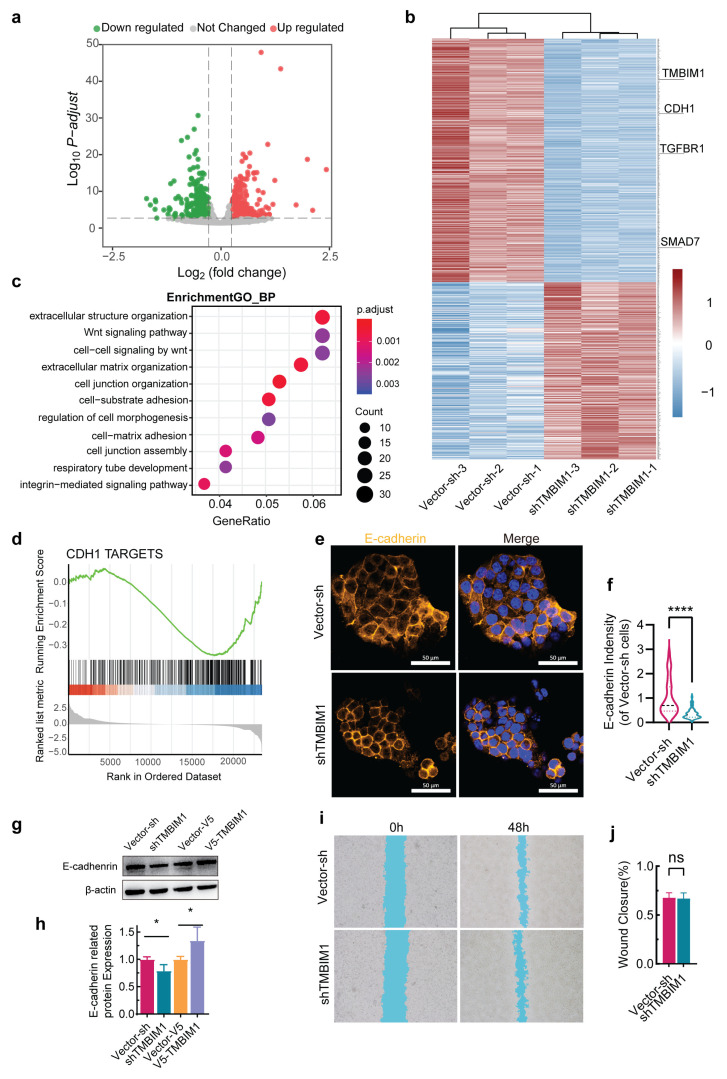
*TMBIM1* knockdown disrupts cell adhesion and alters gene expression in HCT-116 cells. (**a**) Volcano plot illustrating differentially expressed genes in shTMBIM1 cells compared to control. (**b**) Heatmap of differentially expressed genes between shTMBIM1 and control groups. (**c**) Gene Ontology (GO) enrichment analysis of dysregulated pathways. (**d**) Gene Set Enrichment Analysis (GSEA) showing significant changes in E-cadherin (*CDH1*)-related targets. (**e**) Immunofluorescence staining of E-cadherin in control and TMBIM1-knockdown HCT-116 cells. (**f**) Mean intensity of E-cadherin compared to the Vector-sh group (n = 74 for Vector-sh, n = 106 for shTMBIM1). (**g**,**h**) Western blot detection of E-cadherin expression in stable cell lines with altered TMBIM1 expression. (**i**) Wound healing assay from 0 to 48 h in control and TMBIM1-knockdown HCT-116 cells. (**j**) Quantification of wound closure (n = 9 per group). Data represent means ± SD. Statistically significant differences were determined using two-tailed student’s test (**f**,**h**,**j**). ns, not significant; * *p* < 0.05; **** *p* < 0.0001.

To comprehensively characterize the transcriptomic profile of TMBIM1 manipulation in HCT-116 cells, we performed RNA sequencing on HCT-116-shTMBIM1 and overexpression HCT-116-V5-TMBIM1 cell lines, identifying 810 differentially expressed genes (341 upregulated, 469 downregulated) specifically upon TMBIM1 depletion. Bioinformatic analyses including heatmap clustering and volcano plot visualization (Figure 5a,b) revealed pronounced alterations in Wnt signaling and cell–cell junction pathways (Figure 5c), with over-expression failing to elicit similar adhesion-related changes (Supplementary Figure S1b,c), suggesting a unique loss-of-function mechanism.

Western blot validation demonstrated reduced E-cadherin expression in HCT-116-shTMBIM1 cells (Figure 5g,h), which was further supported by immunofluorescence quantification showing a significant reduction in E-cadherin mean density upon TMBIM1 knockdown (Figure 5e,f). These findings were further supported by Gene Set Enrichment Analysis (GSEA) of *CDH1* target genes. Interestingly, despite these adhesion defects, wound healing assays showed no enhanced migration in HCT-116-shTMBIM1 cells (Figure 5i,j). Collectively, these multi-omics data link TMBIM1 knockdown in HCT-116 cells to transcriptional reprogramming of adhesion and Wnt-related pathways, reduced E-cadherin expression, and a consequent shift toward a dispersed cellular organization without increased migratory capacity.

### 2.6. TMBIM1 Depletion Promotes an Aggressive Phenotype by Dysregulating β-Catenin in HCT-116 Cells

**Figure 6:**
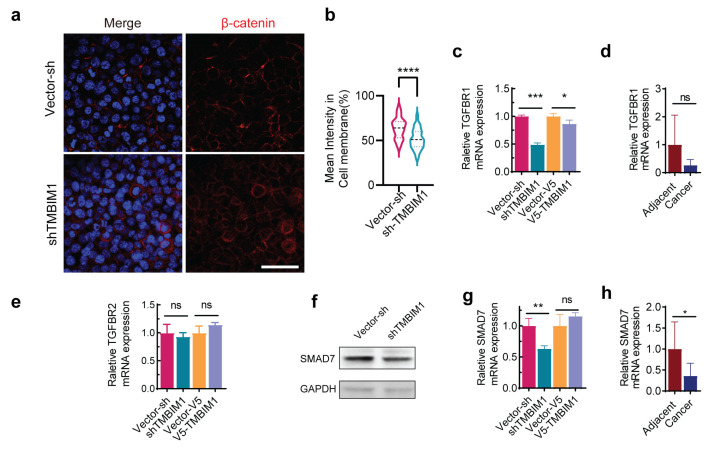
*SMAD7* expression is reduced upon *TMBIM1* knockdown. (**a**,**b**) Immunofluorescence staining of β-catenin in control and *TMBIM1*-knockdown HCT-116 cells (**a**), and quantitative analysis of membrane-localized β-catenin intensity (**b**) (n = 75–76 cells from 4 independent samples per group), scale bar = 50 µm. (**c**) *TGFBR1* mRNA expression in shTMBIM1, V5-TMBIM1, and control cell lines. (**d**) *TGFBR1* mRNA levels in tumor-adjacent tissues and tumor tissues of clinical samples. (**e**) *TGFBR2* mRNA expression in shTMBIM1, V5-TMBIM1, and control cell lines. (**f**) SMAD7 protein level in shTMBIM1 and control cell lines as measured by Western blot. (**g**) *SMAD7* mRNA levels in shTMBIM1, V5-TMBIM1, and control cell lines as measured by qPCR. (**h**) *SMAD7* mRNA levels in tumor-adjacent tissues and tumor tissues of clinical samples (n = 3). Data represent means ± SD. Statistically significant differences were determined using unpaired two-tailed student’s test. ns, not significant; * *p* < 0.05; ** *p* < 0.01; *** *p* < 0.001; **** *p* < 0.0001.

To investigate the mechanism by which TMBIM1 loss alters cell adhesion and enhances viability, we analyzed genes associated with E-cadherin (*CDH1*) signaling. In TMBIM1-knockdown HCT-116 cells, we confirmed a pronounced downregulation of E-cadherin (Figure 5b). This loss of E-cadherin disrupted adherens junctions, as evidenced by the redistribution of β-catenin from the membrane to the cytoplasm (Figure 6a,b). Notably, HCT-116 cells harbor a characteristic S45F mutation in β-catenin that renders it resistant to degradation [22]. Therefore, the E-cadherin-dependent release and cytoplasmic accumulation of this stable β-catenin favors its nuclear translocation, thereby sustaining the transcription of pro-proliferative genes even in the context of partial downregulation of upstream Wnt pathway components (Figure 5c).

We next assessed the impact of TMBIM1 on the TGF-β pathway, a key suppressor of epithelial cell growth. TMBIM1 knockdown significantly reduced *TGFBR1* mRNA expression, while its overexpression increased it (Figure 6f), suggesting that TMBIM1 is a positive regulator of *TGFBR1* expression. This regulatory relationship was selective, as *TGFBR2* levels remained unchanged (Figure 6h). Consistent with reduced TGF-β receptor signaling, the expression of SMAD7, a direct transcriptional target and negative feedback regulator of the pathway, was markedly decreased at both the mRNA and protein levels (Figure 6c,d). The clinical relevance of these findings was supported by analyses of COAD patient data, which revealed concurrent downregulation of both *TGFBR1* and *SMAD7* transcripts in tumor tissues compared to normal counterparts (Figure 6e,g). These results suggest that downregulation of TMBIM1 may contribute to attenuated TGF-β growth-inhibitory signaling in cancer cells, at least in part through reduced TGFBR1 expression.

Collectively, while TMBIM1 knockdown induces morphological changes in NCM460 cells, it triggers a more aggressive phenotype in HCT-116 cells, potentially leveraging their inherent mutations. This phenotype includes profound morphological disruption, loss of cell adhesion, and elevated metabolic activity (Figure S5a). Our data are consistent with a model in which TMBIM1 loss promotes tumorigenicity by coordinately destabilizing cell adhesion (via E-cadherin/β-catenin) and attenuating growth-suppressive signaling (via the TGF-β pathway).

## 3. Discussion

Genetic studies have previously identified the rs992157 variant at the 2q35 locus as a susceptibility factor for colorectal cancer, located within an intron of the *TMBIM1* gene [10]. This locus has been implicated in modulating the risk of COAD and exhibits pleiotropic effects in inflammatory bowel disease. In this study, we confirmed differential expression of TMBIM1 between tumor tissues and matched adjacent normal colorectal tissues. Our results demonstrated significantly lower TMBIM1 expression in COAD tumors compared to normal tissues. This correlation is consistent with the possibility that the risk allele could influence cancer risk through modulating TMBIM1 expression, thereby modestly increasing cancer risk. When combined with environmental factors such as dietary habits, gut microbiota, and microbial metabolites, this effect may further enhance susceptibility to colorectal cancer [23,24].

To elucidate the functional role of TMBIM1, we performed knockdown and knockout experiments in NCM460 cells. TMBIM1 depletion induced marked cellular phenotypes, including disrupted morphology (rounded cell shape, Figure 2n,o), impaired growth, and reduced cell–cell adhesion. These observations underline the importance of TMBIM1 for maintaining the structural integrity of colonic epithelial cells and their intercellular connections. Given that the colonic epithelium relies on tightly regulated cell–cell junctions and mechanical extrusion-based renewal mechanisms [25], TMBIM1 deficiency, by disrupting epithelial morphology and adhesion, could potentially compromise barrier function likely compromises epithelial barrier stability, rendering cells more susceptible to inflammatory stimuli and microenvironmental stressors [23,26,27]. This dysfunction creates a permissive microenvironment for malignant transformation [9]. These findings align with TMBIM1’s protective roles in other disease contexts: knockout mouse models demonstrate its role in preventing age-related vascular degeneration via endosomal/lysosomal remodeling [11], while studies in non-alcoholic fatty liver disease (NAFLD) and pathological cardiac hypertrophy emphasize its function in mediating lysosomal degradation of activated TLR4 [19]. Collectively, TMBIM1 appears to sustain tissue integrity and suppress pathological progression through mechanisms involving cellular morphology maintenance, intercellular connection regulation, and endosome/lysosome pathway participation (e.g., multivesicular body (MVB) formation) [19,28].

Further mechanistic investigations revealed a link between TMBIM1 and E-cadherin. Knockdown of TMBIM1 in HCT-116 cells reduced E-cadherin expression. Both TMBIM1 and E-cadherin are abundantly expressed in the colonic epithelium, whereas forced over-expression of TMBIM1 increased *CDH1* mRNA levels without a corresponding rise in protein abundance (Supplementary Figure S5b,c). Notably, E-cadherin loss directly unleashes β-catenin signaling dysregulation by promoting its nuclear translocation. When this occurs in the colonic crypts, it further disrupts the relatively permissive Wnt signaling regulation characteristic of this compartment [29,30,31]. In HCT-116 cells, which harbor a mutant form of β-catenin, this aberrant signaling is constitutively active, continuously driving pro-oncogenic transcription and enhancing proliferative capacity [32,33], a stark contrast to the regulated degradation in wild-type NCM460 cells. The observed *CDH1* reduction in our study likely suggests that cancer tissue cells may progress toward a more poorly differentiated state, concurrently marked by increasingly prominent nuclear β-catenin positivity that indirectly promotes oncogenic processes. This also indicates that under conditions of aberrant TMBIM1 expression, the impaired degradation efficiency of β-catenin in colonic crypt cells further increases the probability of oncogenic transformation [34,35]. The context-dependent role of TMBIM1, observed specifically in MSI-H/L tumors but not in MSS or unselected cohorts, may be linked to the distinct tumor immune microenvironment or differential apoptotic signaling in these subtypes.

Taken together, our data support a model where TMBIM1 contributes to colonic epithelial integrity. The complex, non-linear relationship between TMBIM1 and E-cadherin expression observed in our study warrants further investigation. Our findings highlight TMBIM1’s potential as a context-dependent factor in CRC, suggesting it merits future exploration for subtype stratification and therapeutic targeting.

## 4. Materials and Methods

### 4.1. Mouse Models

The 4-week-old BALB/c male nude mice were purchased from Vital River (Beijing, China). Six mice per group were randomly allocated, and all animal experiments were conducted by standard procedures and approved by the Laboratory Animal Center, Southeast University. Food and water were offered ad libitum.

HCT-116 cells (5 × 10^6^ in 200 μL PBS) were injected subcutaneously into the left flank of each mouse. Monitored mice daily for any discomfort and weighed every 2 days to monitor the physical condition. Mice were sacrificed after 3 weeks of tumor induction. Tumor volumes were calculated to determine the tumor growth according to the formula (width^2^ × length)/2.

### 4.2. Cell Lines

The human colon epithelial cell line NCM460 and colorectal cancer cell lines HCT116, HT29, LoVo, LS174T, SW480, SW620, HCT8, and SNU-C1 were obtained from the Chinese Academy of Sciences (Shanghai, China). Cells were maintained as follows: NCM460, LoVo, SW480, SW620, and SNU-C1 were cultured in RPMI 1640 medium (Gibco, Waltham, MA, USA, 11875093); HT29 and LS174T were cultured in high-glucose DMEM (Gibco, 11965092); HCT116 cells were cultured in McCoy’s 5A medium (Gibco, 16600082). All media were supplemented with 10% fetal bovine serum (FBS, Gibco, A5256701). Cells were incubated at 37 °C in a humidified atmosphere containing 5% CO_2_.

### 4.3. RNA Isolation and Quantification

Total RNA was extracted from human colon tissues and cultured cell lines using RNAiso reagent (Vazyme, Nanjing, China, #R401-01-AA) according to the manufacturer’s instructions. cDNA was synthesized from the extracted RNA using the HiScript II 1st Strand cDNA Synthesis Kit (Vazyme, #R312-02,). Quantitative real-time PCR (qRT-PCR) was performed using Hieff^®^ qPCR SYBR Green Master Mix (Yeasen, Shanghai, China, #11203ES03). The relative expression of target genes was normalized to that of *GAPDH*. The primer sequences used were as follows: *TMBIM1* Forward: 5′-ACCAAACCAAAGCCGTCATCA-3′; Reverse: 5′-GGAGCCAGTAAACGTATTGGAAG-3′; *GAPDH* (human) Forward: 5′-ACAGTCAGCCGCATCTTC-3′; Reverse: 5′-CCAATACGACCAAATCCGTTG-3′; *SMAD7* (human) Forward: 5′-TGTCCAGATGCTGTGCCTTCCT-3′; Reverse: 5′-CTCGTCTTCTCCTCCCAGTATG-3′.

### 4.4. Lentivirus Production and Cell Transduction

Lentiviral constructs expressing TMBIM1-specific shRNA and the negative control vector (pLKO.1-Puro, Vehicle) were obtained from Miaoling Bio (P0258, Wuhan, China). The targeting sequence for TMBIM1 shRNA was: shTMBIM1: Top strand: 5′-TGCGCCTTTGTGAGGAGAAATGCGAACATTTCTCCTCACAAAGGCGCTTTTTTC-3′;Bottom strand: 5′-TCGAGAAAAAAGCGCCTTTGTGAGGAGAAATGTTCCATTTCTCCTCACAAAGGCGCA-3′. The negative control shRNA sequence was: 5′-TTCTCCGAACGTGTCACGT-3′. For knockdown studies, HCT-116 cells were transduced with lentiviral particles and selected using puromycin-containing medium. Knockdown efficiency was confirmed by qRT–PCR and Western blotting.

For TMBIM1 overexpression, the full-length coding sequence (CDS) of TMBIM1 mRNA was cloned into the BamHI and XhoI sites of the pLenti6/V5-GW/lacZ lentiviral vector (Miaoling Bio). HCT-116 and LS-174t cells were transduced with either TMBIM1-overexpressing or control lentivirus and selected with blasticidin. Over-expression efficiency was validated by qRT–PCR and Western blotting.

### 4.5. TMBIM1 Knockout in NCM460 Cells

The TMBIM1-knockout NCM460 cell line was generated using the CRISPR-Cas9 system. Two single-guide RNAs (sgRNAs) were designed to target sequences within exon 3 of the *TMBIM1* gene: sgRNA1: Forward: 5′-CACCGAAATCACACGCTGCCCTCTA-3′; Reverse: 5′-AAACTAGAGGGCAGCGTGTGATTTC-3′; sgRNA4: Forward: 5′-CACCGCAGGACTCACCGTAGTTCAT-3′; Reverse: 5′-AAACATGAACTACGGTGAGTCCTGC-3′.

These sgRNAs were cloned into the lentiCRISPRv2 vector (Miaoling Bio). The constructed plasmid and helper plasmids were co-transfected into 293FT cells for lentivirus production. Viral supernatant was collected at 24 and 48 h post-transfection and concentrated by ultracentrifugation at 70,000 × g. NCM460 cells were treated with a 1:100 dilution of lentivirus in the culture medium. After 4 days, puromycin was added to select transduced cells for 2 weeks. Positive cells were then trypsinized, diluted to a single-cell suspension, and plated into 96-well plates at a density of ≤1 cell per well. Clones were expanded for 3–4 weeks. Monoclonal colonies were transferred to 6-well plates, and *TMBIM1* knockout was validated by PCR amplification (primers: Forward: 5′-TCTTAAACCACAACAGGGAGTT-3′; Reverse: 5′-AGACAGAAACAGAGGGAGAGA-3′) followed by Sanger sequencing.

### 4.6. Western Blotting

Cell and patient tissue samples were lysed in RIPA buffer supplemented with 1% protease and phosphatase inhibitor cocktail (Sigma-Aldrich, St. Louis, MO, USA). The lysates were centrifuged, and the supernatants were collected for protein quantification. Equal amounts of protein were separated by SDS-polyacrylamide gel electrophoresis and transferred to a PVDF membrane. The membrane was blocked with 5% BSA in TBST for 1 h at room temperature and then incubated with the following primary antibodies at 4 °C overnight: anti-TMBIM1 (Abcam, Cambridge, UK, ab121358; 1:1000), anti-GAPDH (Proteintech, Chicago, IL, USA, #10494-AP; 1:1000), and anti-SMAD7 (Abcam, #ab216428; 1:1000). After washing three times with TBST, the membrane was incubated with species-specific HRP-conjugated secondary antibodies for 1 h at room temperature. Protein bands were visualized using an ECL substrate (#1705061, Bio-Rad, Hercules, CA, USA) and imaged with a ChemiDoc Touch Imaging System (Tanon, Shanghai, China).

### 4.7. Cell Viability Assay

Cells were seeded in 96-well plates. Cell viability was measured at 0, 24, 48, 72 and 96 h using a CCK-8 kit (Dojindo, Kumamoto, Japan, CK04). Briefly, 10 μL of CCK-8 solution was added to each well, and the plates were incubated at 37 °C in the dark for 1.5 h. Absorbance was measured at 450 nm using a microplate reader.

### 4.8. Colony Formation Assay

Stably transfected HCT-116, LS-174t and NCM460 cells with either TMBIM1 knockdown or overexpression were plated in 3.5 cm dishes and cultured in complete medium at 37 °C for 21 days. Cells were then fixed with 4% paraformaldehyde and stained with 2% crystal violet. Images of the resulting colonies were acquired for subsequent analysis.

### 4.9. Cell Cycle Analysis

The cells were digested with trypsin, and Centrifugal collected, then washed twice in PBS and fixed with ice-cold 70% ethanol. Fixed cells were incubated with 50 μg/mL propidium iodide and 100 μg/mL RNase at 37 °C for 20 min and then analyzed with a FACScan flow cytometer (Becton Dickson, Franklin Lakes, NJ, USA).

### 4.10. Apoptosis Assay

An Annexin V-FITC/PI Detection Kit (BD Biosciences, San Diego, CA, USA, 556547) was used for the determination of cell apoptosis. HCT-116 cells were harvested, washed twice with cold PBS, and re-suspended in 1× binding buffer at a concentration of 1 × 10^6^ cells/mL. Subsequently, according to the manufacturer’s instructions, the cells were stained with annexin V-FITC and PI for 15 min at 37 °C. Then, the cells were analyzed immediately using a FACS Calibur flow cytometer (Becton-Dickinson, Fullerton, CA, USA).

### 4.11. Immunofluorescence

Fresh patient-derived tissue samples were prepared as frozen sections (18 μm thick). Sections were washed twice with 1× PBS and blocked with IF blocking buffer for 1 h at room temperature. Subsequently, they were incubated with the following primary antibodies diluted in antibody dilution buffer: anti-TMBIM1 (Abcam, Cambridge, UK, ab121358; 1:100) and anti-α-Tubulin (CST, Danvers, MA, USA, #3873; 1:100) overnight at 4 °C, followed by incubation with corresponding secondary antibodies for 1 h at room temperature.

HCT-116 cells were grown on poly-L-lysine-coated coverslips in 24-well plates. After attachment, cells were fixed with 4% paraformaldehyde for 20 min, permeabilized with 0.25% Triton X-100 for 10 min, and blocked. They were then incubated with primary antibodies against ZO-1 (CST, #13663; 1:200), E-Cadherin (CST, #3195; 1:200), β-Catenin (CST, #8480; 1:100), and TMBIM1 (Abcam, ab121358; 1:100) at 4 °C overnight, followed by secondary antibody incubation for 1 h at room temperature. Coverslips were mounted with Antifade Mounting Medium (Invitrogen, Waltham, MA, USA, P36971) and imaged using a confocal microscope (Zeiss, Jena, Germany, LSM800). Both the cell area and cell circularity were analyzed with the ImageJ software. DiO staining of TMBIM1-knockout and control NCM460 cells was performed according to the manufacturer’s instructions (Beyotime, Shanghai, China C1038). Images were acquired using a fluorescence microscope (Olympus, Tokyo, Japan, BX53), capturing both differential interference contrast (DIC) and DiO fluorescent channels simultaneously.

### 4.12. Immunohistochemistry (IHC)

Tumor tissues were cut into 5-μm-thick sections. Primary antibodies against Ki67 (Abcam, #ab15580, 1:200), TMBIM1 (Affinity, Xuzhou, China, #DF4580, 1:100) were used. For visualization, the complex was visualized with DAB complex, and the nuclei were counterstained with hematoxylin.

### 4.13. Patient Datasets and Bioinformatics Analysis

Transcriptomic data and clinical information for colon adenocarcinoma (COAD) were obtained from The Cancer Genome Atlas (TCGA) portal. This study analyzed 275 primary COAD tumors and 41 matched normal tissue samples. Survival analysis within the microsatellite instability-high/low (MSI-H/L) subgroup (Figure 1c) was performed on 94 patients (47 with high TMBIM1 expression and 47 with low expression). Detailed clinical annotations for the TCGA-COAD cohort are publicly accessible and can be explored interactively via the UALCAN online tool.

### 4.14. Statistical Analysis

All data presented are representative of at least three independent experiments. Statistical analyses were performed using GraphPad Prism (version 9.4.1). For comparisons among more than two groups, one-way ANOVA with Dunnett’s multiple comparisons test was applied. Comparisons between two groups were conducted using two-tailed Student’s *t*-tests. The specific statistical test used in each experiment is detailed in the corresponding figure legend. Data are expressed as mean ± SD unless otherwise stated. A *p*-value of less than 0.05 was considered statistically significant (* *p* < 0.05, ** *p* < 0.01, *** *p* < 0.001, **** *p* < 0.0001).

## Data Availability

The data presented in this study are available in this article. The raw sequencing data generated in this study had deposited in SRA (accession number PRJNA1394857).

## Supplementary Materials

The following supporting information can be downloaded at https://www.mdpi.com/article/10.3390/ijms27021090/s1.

## Abbreviations

The following abbreviations are used in this manuscript:

TMBIM1	Transmembrane BAX Inhibitor Motif-Containing 1
COAD	Colon Adenocarcinoma
READ	Rectum Adenocacinoma
CRC	Colorectal Cancer
MSI-H	Microsatellite Instability-High
MSI-L	Microsatellite Instability-Low
MSS	Microsatellite Stable
*CDH1*	E-Cadherin Gene
TGF-β	Transforming Growth Factor Beta
TGFBR1/2	Transforming Growth Factor Beta Receptor 1/2
ZO-1	Zonula Occludens-1
