# Supplementary material for: Loss of Epithelial Homeostasis Driven by TMBIM1 Depletion via E-Cadherin Junction Disassembly
Source: Int J Mol Sci. 2026 Jan 22;27(2):1090. doi: 10.3390/ijms27021090 (PMC12842013; doi:10.3390/ijms27021090)
Supplement: Supplementary file 1 [file ijms-27-01090-s001.zip › ijms-4009914-SI.pdf]

Supplementary Figure S1. Analysis of TMBIM1 expression and its prognostic value in rectal adenocarcinoma (READ).

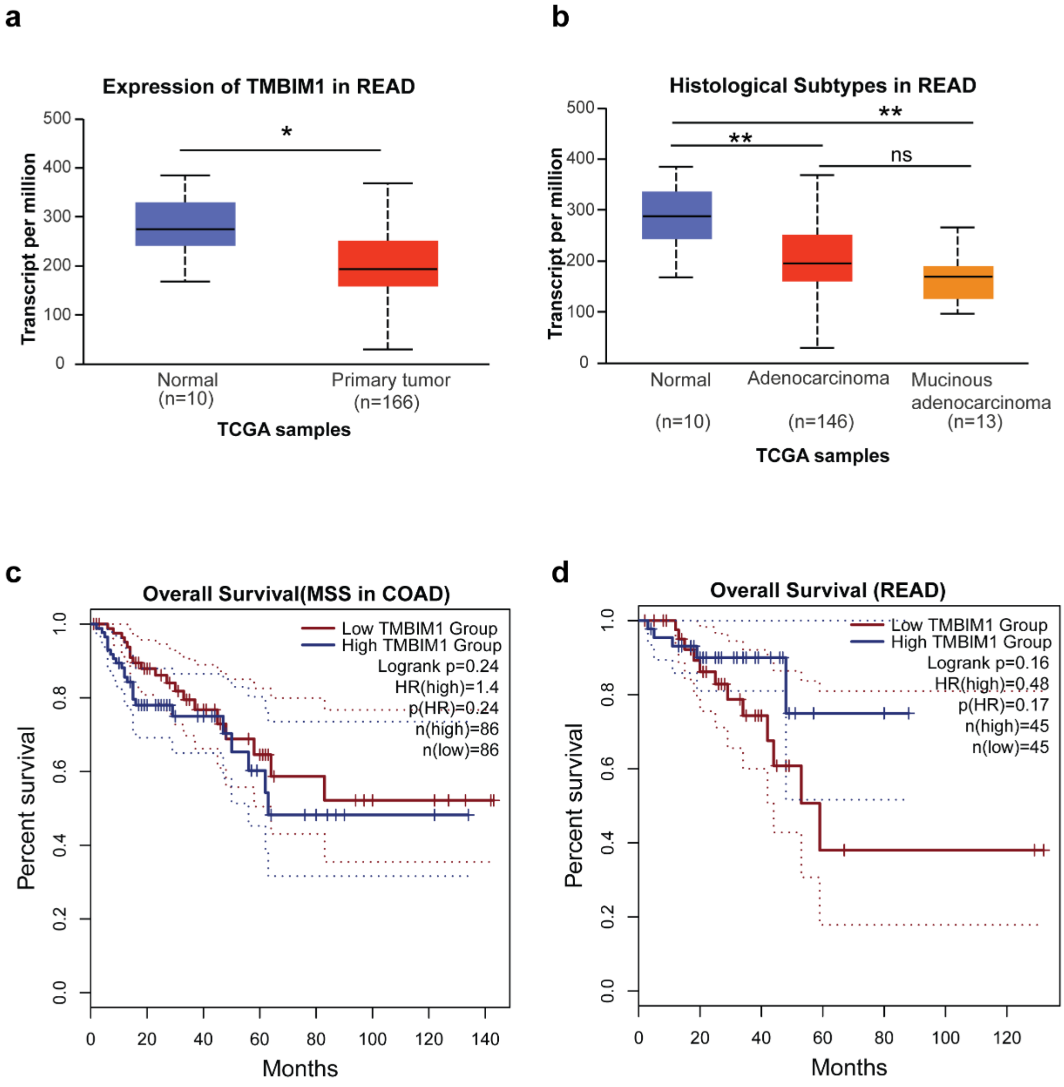

(a) Analysis of TMBIM1 mRNA expression between healthy individuals (n=10) and READ patients (n=166) based on TCGA data ($P < 0.05$).

(b) Comparison of TMBIM1 expression across normal rectal tissues (n=10), conventional READ (n=146), and mucinous READ (n=13) subtypes. Statistical significance is indicated as: Normal vs. READ, $P < 0.001$; Normal vs. mucinous READ, $P < 0.001$; READ vs. mucinous READ, not significant (ns).

(c) Kaplan-Meier survival analysis of microsatellite stable (MSS) colon cancer patients stratified by TMBIM1 expression (n=86 per group, log-rank p = 0.24).

(d) Kaplan-Meier survival analysis of the overall READ patient cohort stratified by TMBIM1 expression (n=45 per group, log-rank p = 0.16).

For survival analyses (c and d), TMBIM1 expression was normalized to *ACTB*, and analysis was performed via GEPIA2 based on TCGA data. Significance levels: $P < 0.05$; *$P < 0.01$; **$P < 0.001$; ***$P < 0.0001$.

Supplementary Figure S2. Validation of TMBIM1 genetic manipulation in normal colon epithelial NCM460 cells.

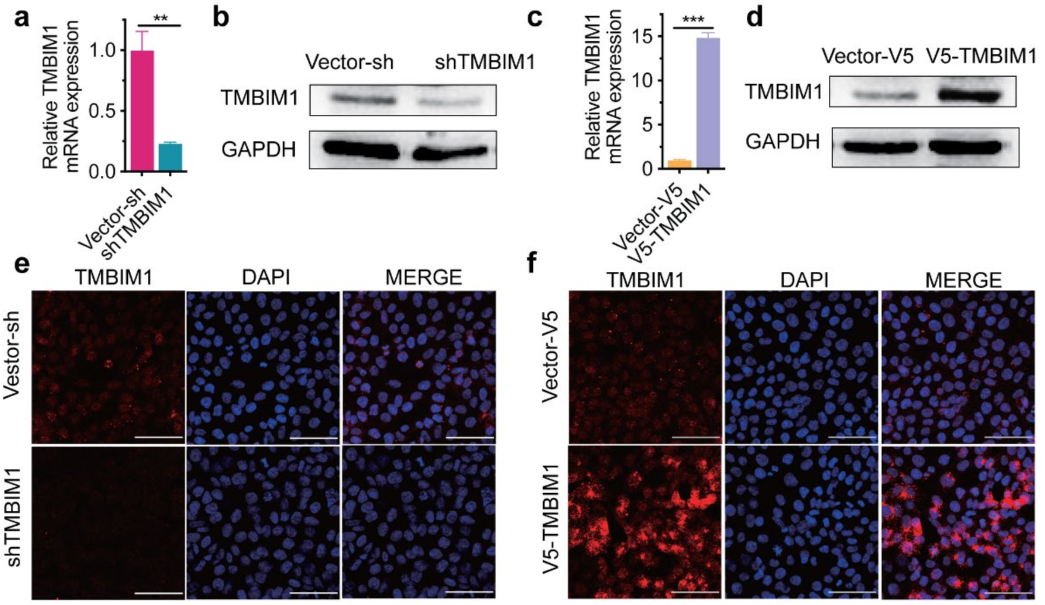

(a) Crystal violet staining of single colonies in Vector-sh, shTMBIM1, empty vector control (Vector-V5), and TMBIM1-overexpressing (V5-TMBIM1) NCM460 cells, scale bar=50 μm.

(b) Schematic representation of the lentivirus-mediated TMBIM1 knockout strategy in NCM460 cells.

(c) The clones of TMBIM1 knockout NCM460 cells. Clone A, wild-type (+/+); Clone B, heterozygous knockout (+/–); Clone C, homozygous knockout (–/–). (d Western blot analysis confirming TMBIM1 protein expression levels in wild-type NCM460 cells and the knockout clones (Clone A, B, and C).

Supplementary Figure S3. Validation of TMBIM1 knockdown and overexpression in HCT-116 cells.

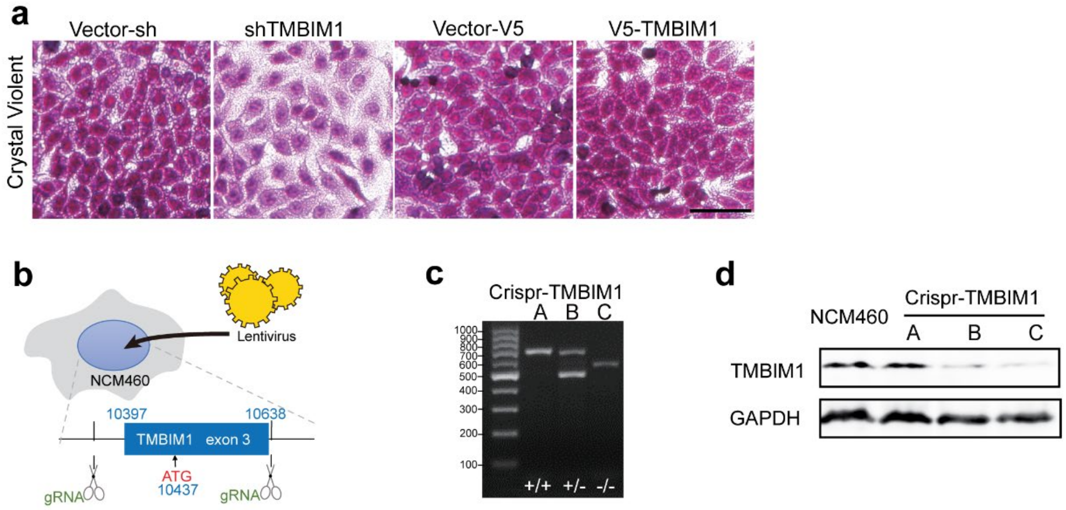

(a) Relative TMBIM1 mRNA expression in shTMBIM1 and control cells (n = 3 per group).

(b) Western blot analysis of TMBIM1 protein levels in control and shTMBIM1 HCT-116 cells.

(c) Relative TMBIM1 mRNA expression in V5-TMBIM1 and control cells (n = 3 per group).

(d) Western blot of TMBIM1 protein in control and V5-TMBIM1 HCT-116 cells.

(e, f) Immunofluorescence staining of TMBIM1 in shTMBIM1 and V5-TMBIM1 HCT-116 cells; scale bar = 50 μm.

Statistical significance was determined unpaired two-tailed Student's t-test. Significance levels are indicated as $*P < 0.05$; $**P < 0.01$; $***P < 0.001$; $****P < 0.0001$.

Supplementary Figure S4. Validation of tumor-suppressive phenotypes upon TMBIM1 manipulation in LS174T cells.

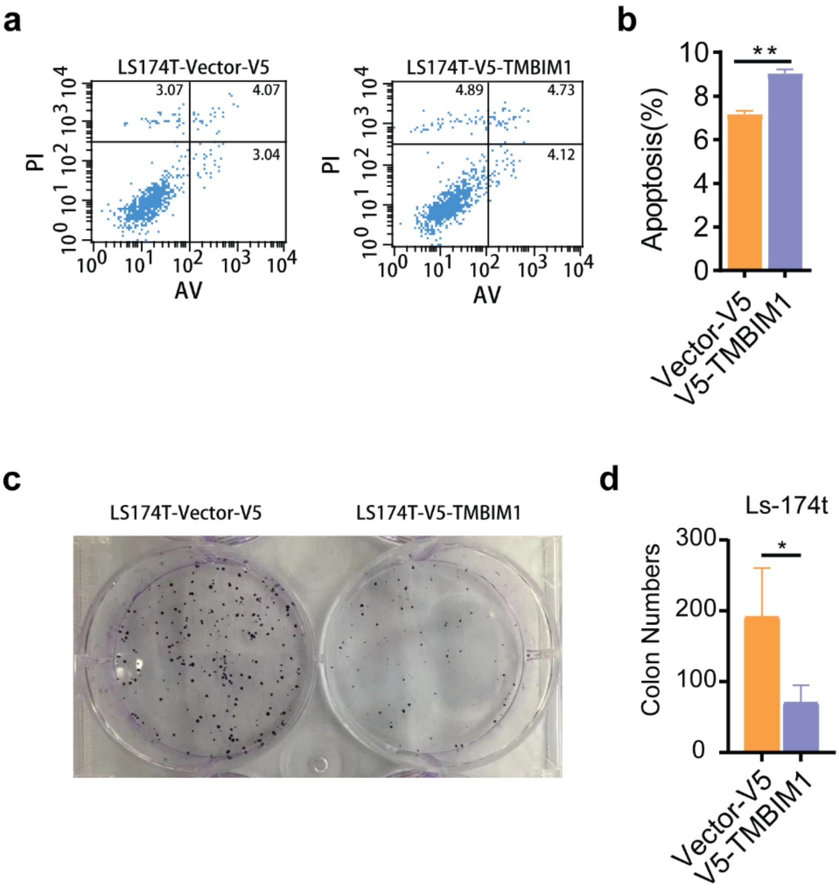

(a, b) Apoptosis analysis in LS174T cells. Representative flow cytometry plots **(a)** and quantification **(b)** showing the percentage of apoptotic cells in shTMBIM1 knockdown groups compared to Vector-sh control groups (n = 3 independent experiments). Data are presented as mean ± SD.

(c, d) Colony formation assay in LS174T cells. Representative images of crystal violet-stained colonies **(c)** and quantification **(d)** in TMBIM1-overexpressing (V5-TMBIM1) cells compared to empty vector controls (n = 3 independent experiments). Data are presented as mean ± SD.

Statistical significance was determined unpaired two-tailed Student's t-test. Significance levels are indicated as $*P < 0.05$; $**P < 0.01$; $***P < 0.001$; $****P < 0.0001$.

Supplementary Figure S5. Mild transcriptional alterations upon TMBIM1 overexpression in HCT-116 cells

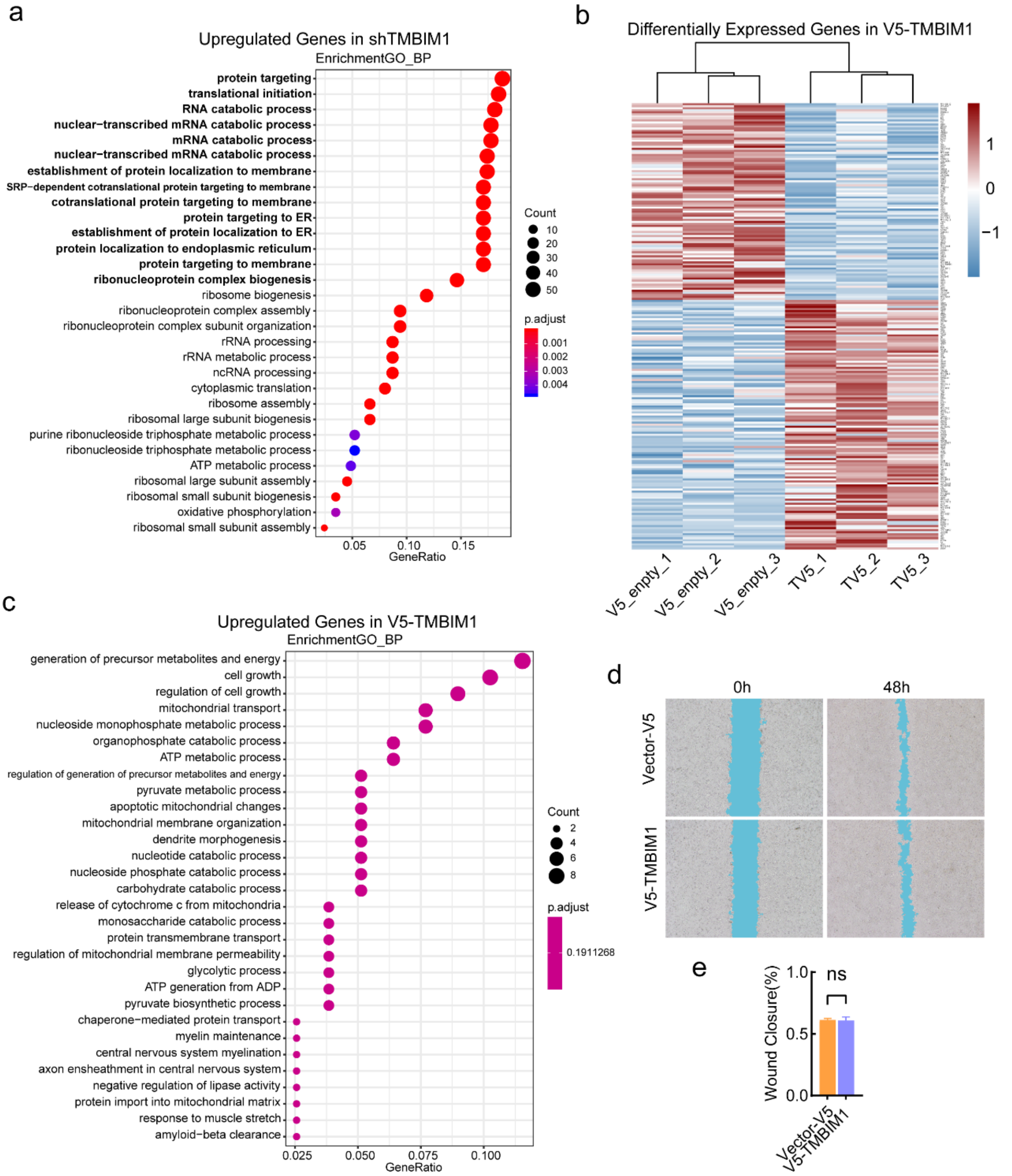

(a) Gene Ontology (GO) enrichment analysis of biological processes up-regulated in TMBIM1-knockdown cells.

(b) Heatmap of differentially expressed genes between V5-TMBIM1 overexpression and control groups.

(c) GO enrichment analysis of biological processes up-regulated in TMBIM1-overexpressing cells.

(d) Wound healing assay from 0 to 48 hours in control and TMBIM1-overexpressing HCT-116 cells.

(e) Quantification of wound closure rate in V5-TMBIM1 and control groups (n = 9 per group).

Data represent means ± SD. Statistically significant differences were determined using two-tailed student's test(e).$^{*}P < 0.05$; $^{**}P < 0.01$; $^{****}P < 0.0001$.
